# How do students' perceptions of research and approaches to learning change in undergraduate research?

**DOI:** 10.5116/ijme.5523.2b9e

**Published:** 2015-04-12

**Authors:** Rintaro Imafuku, Takuya Saiki, Chihiro Kawakami, Yasuyuki Suzuki

**Affiliations:** 1 Medical Education Development Center, Gifu University, Gifu, Japan

**Keywords:** undergraduate research, perceptions of re-search, approaches to learning, continuing professional development

## Abstract

**Objectives:**

This study aimed to examine how students' perceptions of research and learning change through participation in undergraduate research and to identify the factors that affect the process of their engagement in re-search projects.

**Methods:**

This qualitative study has drawn on phenomenography as research methodology to explore third-year medical students' experiences of undergraduate research from participants' perspectives (n=14). Data included semi-structured individual interviews conducted as pre and post reflections. Thematic analysis of pre-course interviews combined with researcher-participant observations in-formed design of end-of-course interview questions.

**Results:**

Phenomenographic data analysis demonstrated qualitative changes in students' perceptions of research. At the beginning of the course, the majority of students ex-pressed a relatively narrow definition of research, focusing on the content and outcomes of scientific research. End-of-course reflections indicated increased attention to research processes including researcher autonomy, collaboration and knowledge construction processes. Furthermore, acknowledgement of the linkage between research and learning processes indicated an epistemological change leading them to take a deep approach to learning in undergraduate research. Themes included: an inquiring mind, synthesis of knowledge, active participation, collaborative and reflective learning. However, they also encountered some difficulties in undertaking group research projects. These were attributed to their prior learning experiences, differences in valuing towards interpersonal communication, understanding of the research process, and social relationships with others.

**Conclusions:**

This study provided insights into the potential for undergraduate research in medical education. Medical students' awareness of the linkage between research and learning may be one of the most important outcomes in the undergraduate research process.

## Introduction

Research activity is considered one of the high-impact educational practices in that the vital skills and attitude for lifelong learners can be cultivated through inquiry.^1^^-^^3^ Undergraduate research was defined as any teaching and learning activity in which undergraduate students are actively engaged with the research content, process or problems of their discipline.^4^ That is, research is not merely pursuit of academic career and advancement of knowledge (i.e., content). Rather, it also includes an aspect of learning process.^5^^-^^7^Development of research skills is also important in health professions education.^8^^,^^9^

Research activities by undergraduates are a powerful way of enhancing medical students' basic skills and attitude necessary for future professional practice. Inquiry and an evidence-based medicine (EBM) approach are complimentary processes in that they include recognition of important questions, search for the best research evidence, critical appraisal of the evidence, and application of the evidence to practice.^10^^-^^12^ Modern clinicians, therefore, have to understand both the principles of research and how evidence is derived.^13^

Integration of EBM elements into the undergraduate medical curriculum now has increasing significance. For instance, in the first edition of *Tomorrow's Doctors *issued in 1993, the General Medical Council (GMC) urged innovation in UK undergraduate medical curricula in order to reduce direct instruction of factual content and provide more inquiry-based, student-centred learning environments.^8^ One radical change was the introduction of extensive student choice of study modules, which is currently termed 'student selected components' (SSCs). Basically, these curricula provide medical students with opportunities to select study areas of interest and to pursue what they want to know through inquiry. This can potentially be pedagogically effective vehicles for critical appraisal and research skill development.^13^^,^^14^ Likewise, in Japan, 63 out of 80 medical schools have implemented a research-based course in the undergraduate curriculum.^15^ Although the duration, study area and assessment method are different among the schools, the common educational purpose is to provide opportunities leading to the development of research skills and basic skills necessary to continuing professional development.

Although research activity as an educational practice has been increasingly employed in a variety of disciplines as well as in diverse cultural contexts, students might take different preferred approaches to learning across cultures.^16^^-^^19^For instance, Asian students have been portrayed as typically passive, uncritical and rote learners. Asian students' strong perceptions of teachers as knowledge providers are considered one of the influential factors that affect their passive participation in a classroom.^16^ On the other hand, there is a paradox between such a description of Asian learners and their academic attainment.^17^^-^^19^ Marton and Dall'Alba indicated the qualitatively different ways of experiencing learning in different cultural contexts.^19^ Given the variation in ways individuals experience various phenomena, it is important to understand how Asian learners participate in a student-centred learning environment. As students need to undertake a collective research project in this study, mutual engagement is essential to the process of undergraduate research.

While there is a plethora of discussions on learning outcomes in undergraduate research based on the findings underpinned by a quantitative research paradigm, few studies have examined epistemological changes in research and learning through qualitative analysis of students' research activity.^13^ Therefore, this study was undertaken as an initial investigation into this area. A better understanding of students' research experiences from an emic viewpoint allows educators to clearly identify why and how research activity promotes meaningful learning. Furthermore, identifying factors that affect students' research activity can provide important practical implications to effectively encourage and facilitate research opportunities for students. In order to make contribution to this gap in the literature, this study aimed to investigate 1) how students' perceptions of research and learning change through participation in undergraduate research; and 2) what factors affect the process of their engagement in undergraduate research.

## Methods

### Research approach

Qualitative research methods were used for this study, which was particularly underpinned by phenomenography. The rationale of drawing on phenomenography in this study is that the changes in people's ways of interpreting the nature of research generally take place through their own experience of research and interaction with others.^20^ Phenomenography allows an examination of "the ways in which people experience, conceptualise, perceive, and understand a phenomenon from their own perspectives."^21^ Investigations with a phenomenographic orientation thus focus more on exploration of what is experienced and howit is experienced (i.e., "second-order perspective") than description of the world itself (i.e., "first-order perspective").^17^^,^^20^^,^^21^ Within this setting, students' learning processes in undergraduate research are not analysed in terms of what students learned or remembered but rather attends to the relationship between students and the phenomenon. In particular, we briefly describe how phenomenography interprets a relationship between students' approaches to learning.

Phenomenography enables a mapping of the qualitatively different approaches to learning that students adopt. Students' approaches to learning are not regarded as the personality traits or fixed learning styles, but determined through interaction of a student with a specific learning context.^22^ Phenomenographers have identified three main types of approaches to learning: deep, surface and strategic approaches.^22^^-^^24^ A deep approach to learning involves relating new ideas to previous knowledge and examining the logic of the argument critically, and leads to understanding and long-term retention of concepts. Thus, learners who take a deep approach to learning primarily focus on seeking meaning. In contrast, a surface approach to learning is associated with information reproducing. For instance, students who take a surface approach to learning try to make use of rote learning, memorize information needed for assessment, take a narrow view and concentrate on detail. A strategic approach to learning is taken to obtain high grades and other rewards. The learning strategies of this approach include identifying the assessment criteria and estimating the learning effort, following up all suggested readings, and using previous exam paper to predict questions. Although deep and surface approaches are mutually exclusive, a strategic approach can be linked to either, that is, surface-strategic or deep-strategic approach. These three pre-identified categories were examined simultaneously with the more inductive labelling process.

### Research site

Generally, Japanese medical schools have a 6-year undergraduate curriculum which consists of general education (the first year), pre-clinical studies (the middle 2.5-3 years), and clinical clerkships and preparation for national board examinations (the last 2 years). Sixty out of 80 Japanese medical schools implement a research-based course in the pre-clinical study periods.^15^

The context of this study was Gifu University School of Medicine. There was a mandatory course of "Research Experience" in which all third-year students (n=106) selected a 10-week subject or two 5-week subjects from 23 research themes of basic, social or clinical medical sciences, such as anatomy, legal medicine and paediatrics, and then pursued a research topic of interest. It predominantly involved project work, and there were no other classes during the 'research' weeks to detract from their learning experiences through inquiry. As a summative assessment, they were required to give poster and oral group presentations in front of all third-year students and faculty in the final week.

Medical education research was a 5-week course in 2013, and was altered to a 10-week course in 2014. Class meetings (2-3 hours) were basically scheduled three days a week, and the rest of class time in a week (21 hours) was allotted to self-directed research activity. In every class, students were encouraged to discuss research design and to give a progress report. Academic staff in medical education centre participated as mentors who encourage students to undertake a research project as autonomously as possible.

### Participants

A purposive sampling was adopted, and 14 third-year medical students (9 male, 5 female: S1-S14) who selected medical education research in 2013 or 2014 agreed to participate in this study. They conducted medical education research projects about medical students' perceptions of career choices, learning experiences in PBL tutorials, or gender differences in perceptions of career and family among students. S1 has had some research experience as a student research assistant and S8 holds a Master's degree in psychology. However, the rest of participants has little experience in conducting research.

 In order to achieve the consistency of research context, we carried out data collection only in the medical education centre, because the course structure varied according to the research field, such as the role of tutor and duration/frequency of class meeting. This study was approved by the Institutional Review Board at Gifu University.

### Data collection

This qualitative study drew upon methods of direct observation and semi-structured interviews. Observations allow for insight into contexts, relationships and behaviour by better understanding what participants do. The first author as a participant observer in the two academic years of the study made records to capture the details of students' participation in the course. To deal with observer effect, we did not reveal the specific focus of the observation, but obtained students' consent to observe their learning activities. These observational data were rigorously analysed to gain emic understandings of the context of student research activity and to inform the development of the second (post) interview guide as to more closely situate it to each individual's context.

Each participant was invited to be interviewed twice during the course. The first interview (pre) was conducted in the second week of the course, and the second interview (post) was in the final (fifth or tenth) week of the course. Each interview lasted around 25 to 45 minutes, and was audio-recorded. Japanese transcripts were translated into English by the first author.

In the first interviews, we attempted to elicit information on students' prior experiences, perceptions of research, understanding of student roles and on-going experiences in this programme. Prior research findings in phenomenography have informed the first interview schedule.^22^^,^^25^ In the second, follow-up interviews, we focused on eliciting information on challenges which they found in the process of undertaking a group research project; their approaches to researching; perceived benefits of undergraduate research; and; changes in their perceptions of research and learning. Moreover, we also asked some questions which were informed by the observational data (e.g., *I felt you participated more actively than before. Why did your participation pattern change over time?*).

### Data analysis procedures

Interview data were qualitatively analysed based on the principles of phenomenography as an empirical approach to describing the qualitatively different means of people's experience.^19^^, ^^21^ There are seven common steps of data analysis in phenomenography.^26^ The first step is *familiarization* in which the researchers need to read through transcripts to become familiar with empirical data and obtain a sense of the whole. The second step involves *compilation *ofanswers from all respondents to a certain question. The most significant elements in the answer need to be identified here. The third step is a *condensation* of the individual answers to find the central part of longer answers. The fourth step contains a preliminary *grouping*,and the researchers allocate answers expressing similar ways of understanding the phenomenon to the same category. The fifth step is a preliminary *comparison* of categories with regard to similarities and differences. The sixth step consists of *labelling *to express the core meaning of the category. The seventh step is a *contrastive comparison *of categories. Comparing the categories through a contrastive procedure, the unique character of the categories and its relationship between them are described.

Following these steps above, the data were carefully reviewed multiple times by the research team, and we inductively generated salient categories. In this process, peer debriefing was used as a technique to establish credibility and validity of the data analysis. That is, the authors worked together on the coding of data to prevent some critical problems of analysis, such as misinterpretation of data and vague descriptions of coding. Member checking was also undertaken to confirm whether researchers' interpretation of interview data was congruent with what participants intended to express.

## Results

### Changes in perceptions of research

We examined Japanese medical students' reflection on and perceptions of their experiences in undergraduate research. In particular, the focus of the data analysis was on understanding how students' perceptions of research changed through their experiences of conducting research a project and how the change in epistemological belief regarding research relates to students' approaches to learning. The labelling procedures (see below) produced two core categories, 'content-oriented' and 'process-oriented' approaches to study. Contrastive comparison indicated that, of the total number of 14 students, 10 students' perceptions of research (S1, S2, S3, S4, S5, S7, S8, S10, S11, and S14) qualitatively changed from 'content-oriented' to 'process-oriented' ones during the undergraduate research course. The remaining four students' perceptions of research (S6, S9, S12, and S13) remained 'content-oriented'. In what follows, we detail the establishment of these categories and the result of contrastive comparison.

#### Students' perceptions of research in Week 2

As to students' perception of research at the early stage of the programme, three main themes emerged from the first (pre) interview data: irrelevant activity to undergraduates' life, research methods and outcomes (Table 1). In the first interview, the majority of students expressed a relatively narrow definition of research, focusing on the content and outcomes of scientific research.

In response to what students mean by research in the first interview, some students were not clear about what research is due to their less experience of conducting research. They said that research was irrelevant activity to undergraduates' life in that research tended to be regarded as an activity only by people who pursue academic career, such as postgraduate-level and faculty-level:

"I don't feel familiar with research activity because I haven't conducted it. Since I think research is an activity for becoming academics, I'm not really interested in conducting research, and it is irrelevant to medical students, who want to actively take part in clinical practice, not research position, in the future, like me." (S2, Group 1, Week 2, 2013)

In addition, medical students in this study conceived research as experiment, hypothesis testing or lonely activity of scientists. The term "research" tended to give the medical students a certain impression in association with science experiment.

"In my understanding, research is to make a microscopic study in a scientific laboratory all the day. There is no chance to communicate with others. So, I have a negative impression that research is a lonely activity." (S6, Group 2, Week 2, 2013)

"I think that research means verifying hypotheses through repeated same experiments. So, it is conducted so as to reveal the truth logically based on objective data. I feel it boring and time consuming." (S13, Group 3, Week 2, 2014)

Lastly, their perception of research was related to its outcome and output. Students emphasized "an epoch-making discovery" and "advancement of knowledge" as keywords regarding research. They tended to regard research as scientists' activity which presents new perspective of a certain study field and solution to complex problems. That is, at the early stage of the research project course, the majority of participants in this study thought that undergraduates' life was unrelated to research involvement:

"Research is conducted in order to advance knowledge in your academic field, such as medical sciences. I think publication of journal article and conference presentation can be central to research activity." (S10, Group 3, Week 2, 2014)

"For me, research means discovery of what nobody knows or invention of new devices. I haven't conducted research before, so it's just my impression, but I research works would be achieved by the limited great figures, experts, in academic fields." (S12, Group 3, Week 2, 2014)

**Table 1:** Summary of students' perceptions of research in Week 2

Themes	Codes
Irrelevant activity to undergraduates' life	Unfamiliar activity
	No appeal
Research Methods	Experiment
	Hypothesis testing
	Data gathering
Research outcomes	Advancement of knowledge
	New attempt and discovery of truth
	Solutions to problems
	Academics' activity

#### Students' perceptions of research in final week (tenth or fifth week)

Data analysis of the second (post) interview showed their increased attention to research processes, including autonomy, collaborative working and knowledge construction processes. Furthermore, through participation in research project, they realised that research has something to do with learning process in their own context of studying at the medical school. That is, their perceptions of research were related to experiment, solitude and exhaustive work in Week 2, whereas they could view research as social and cognitive processes of daily activity in the final week. Table 2 provides a summary of their perceptions of research in the final week.

As shown in Table 2, students recognised the linkage of research and learning through participation in research project. In Week 2, they perceived research and learning as separate activities. Specifically, research was viewed as an irrelevant activity to undergraduates' life and also as an activity which was undertaken only by someone who pursues an academic career. However, they began to relate the process of research to their learning experiences during the course. S2 said:

"Recently, I gradually became aware that the processes of learning might be similar to those of research. Like, it includes investigation of what I want to know, information gathering necessary to the goals, and a study meeting with my friends outside classroom. I think what I did in our research project was exactly congruent to the process of learning." (S2, Group 1, Final week, 2013)

In addition to students' awareness of linkage between learning and research processes, research could be viewed as process of inquiry by them in the final week. Students said that researcher autonomy or inquiring mind is a core concept of research. S11 emphasised the importance of inquiring mind in doing research:

"Research is a process of approaching to the truth, which is driven by your inquiring mind. For example, we researched students' perceptions of career choice as medical doctor and family, umm, sharing housework with partner. People's perceptions vary according to their background, and we couldn't draw one definite conclusion from data. However, I really enjoyed working in this process, and my inquiring mind made me participate more actively in the research project." (S11, Group 3, Final week, 2014)

S2 said that researcher’s autonomy during the research process is important for inquiry. Moreover, he noticed that research could be seen as not a special activity of scientist but a daily activity of people.

"Investigating on your own initiative is pivotal to conducting a research project. So, research is to investigate what I want to know on my initiative. In the first interview, I said I had no idea about research, but now I feel research can include not only scientists' work but also our daily activity of learning." (S2, Group 1, Final week, 2013)

As students in this study were engaged in research work as a group, some participants viewed research from a social, interpersonal perspective. In the first interview, research was seen as scientist's lonely activity, whereas students mentioned in the second interview that research was collaborative work. S3 said:

"All group members needed collaboratively work to find out our own way to attain the shared goal of the research project. It is important for each member to actively make contribution to the research project. I needed to understand how I could contribute to group work, like my own role in this group. Before I participated in this course, I thought research should be done alone, but now I realize that research also includes group work, and collaborative work with members is really essential to the research project." (S3, Group1; Final week 2013)

From a cognitive perspective, research was viewed as a knowledge construction process by them in the final week. S14 said:

"I could realise that research includes the processes of integrating a variety of data into the meaning, and presenting the findings to people. It was very difficult to answer our research questions based on such an extensive data obtained from interview and questionnaire survey and we struggled to interpret those data, but I noticed that this process of thinking was research." (S14, Group 3, Final week, 2014)

Therefore, analysis of interview data shows that students' perceptions of research have changed qualitatively through experience in conducting research.

**Table 2:** Summary of students' perceptions of research in the final week

Themes	Codes
Linkage of research and learning	Pursuing a subject of interest
	Process of understanding the reality
	Extension of learning activity
Motivation and autonomy	An inquiring mind
	Active contribution
Collaborative working process	Mutual engagement
	Identity as a member
Knowledge construction process	Synthesis of evidence
	Identification of the principle

### Relationship between perceptions of research and approaches to learning

#### Deep approach to learning

Students who could have a process-oriented perception of research took a qualitatively deeper approach to learning during the course. Five themes regarding deep approach to learning emerged from the analysis of interview data: inquiring mind, synthesis of knowledge, active participation, collaborative learning and reflective learning.

Firstly, their inquiring mind intrinsically motivated their engagement with the research. As S4 mentioned, he did not feel that he was forced to do the research project by someone in a mandatory course. Such motivation has led to their deep approaches to learning.

"I have a research stance that seeks what I want to know for its own sake. Now, I'm not reluctantly doing research under someone's instruction. Rather, with tutor's advices, I'm carrying out the research on my own initiative, umm, pursue what I want to know for my own sake." (S4, Group1; Final week 2013)

"I want to do further investigation by interviewing with the students which can be useful for a deeper analysis of PBL. I felt only questionnaire was not enough to better understand their attitudes toward PBL." (S7, Group 2; Final week 2013)

Secondly, students expressed synthesis of knowledge which is a more complex cognitive process in Bloom's taxonomy.^27^ For instance, S11 fully enjoyed drawing a conclusion from a large amount of data. S11's comments implied that interpretation of the phenomenon involved comparison, integration and categorisation of data:

"I felt really interesting in interpreting the common or different ideas on family and career planning among male and female medical students from extensive data obtained through interviews and questionnaire survey. Apparently, I supposed that those data were not interrelated, but, in fact, I realized that there was a story on what human being is behind the data." (S11, Group 3, Final week, 2014)

Thirdly, in transition from direct instructional context to student-centred learning context, necessity of active learning was strongly acknowledged by them. In doing research project, students needed to collect and analyse data on their own initiative in order to investigate what they want to know. S4 said:

"I've got used to obtaining knowledge by listening to teachers since I was a child. It was a kind of first time to work out a plan for the research project by ourselves. I realised the importance of actively study something in my career as a medical doctor through research design, data collection and analysis in the course." (S4, Group1, Final week, 2013)

Fourthly, in the context of collaborative work, the importance of teamwork was also emphasised. Although S3 felt it difficult to make contribution to the group work, she realised that sharing her opinions can be essential to elaboration of research planning and data analysis in group.

"Through research, I realised the importance of expressing my opinion explicitly. At the beginning, I hesitated to do it, because I worried if my opinion would be off the point in the group discussion, but now I can say any opinions can contribute to the research work, which can be also related to teamwork." (S3, Group 1, Final week, 2013)

Lastly, each student continuously reflected on the progress of their research work, their underlying belief on research and areas of improvement in order to attain the shared goals. For instance, S5 attempted to better understand the nature of qualitative research during this course, and he noticed that this reflective process actually led to his meaningful learning.

"During the research project, I was always thinking of what qualitative research is and how I could qualitatively analyse data obtained. This kind of reflection on what I did and repeatedly thinking of qualitative research connected to meaningful learning. Although I couldn't find out the exact answer during this course, this was a good experience for me." (S7, Group 2, Final week, 2013)

S1 tried to improve the consistency of their work through reflection on the research purposes which were discussed at the early stage. S1 acknowledged the importance of reflection in doing research:

"It was very important to take the consistency of the research into account. Don't forget what we originally wanted to know and clarify. When I was stuck with research planning and data analysis, I always reflected on what we discussed with respect to research questions in Week 1." (S1, Group 1; Final week, 2013)

#### Strategic approach to learning

Students who had only a content-oriented perception of research tended to take a strategic approach to learning, which aims to earn the (highest possible) grades of the course, such as well organised study methods and effective time management.^23^ However, there was a slight change from surface-strategic to deep-strategic approaches. For instance, at the early stage of research project, S6 attempted to manage what he needed to do in his group by minimal effort and only followed tutors' suggestions. S6 stated:

"I felt my ideas were not insightful, and I couldn't effectively make contribution to the group work. That's why I focused on just listening to others and following others' suggestions, which was the most efficient way of completing the task." (S6, Group 2; Week 2, 2013)

His main focus was on finding an efficient way of completing the task in this course. He did not build knowledge through active interaction with members but keep quiet to avoid interrupting others’ discussions. However, as he experienced a group situation where others were stuck and there was frequent silence during research planning, his approach to research project appeared to change to a deep-strategic approach. S6 stated:

"I started to think that I had to share my opinions in our group, otherwise we couldn't finish this project on time.

When they were completely stuck in the meeting, I strongly felt that I needed to do something. Once I made contribution in the discussion, I started to enjoy participating in this project." (S6, Group 2; Final week, 2013)

Although his main aim was to complete the task and to obtain safe grade in this research course, he could take a leadership in the group and share his opinions more actively.

### Factors affecting students' engagement with undergraduate research

This study identified four main inclusive factors which led to the Japanese medical students in this study expressing practical difficulties in the course:

- Prior learning experience
- Values towards interpersonal communication
- Understanding of research process
- Social relationships with tutors and peers

The first factor is their prior learning experiences. Students identified a gap in the instructional approaches between their prior learning experiences and undergraduate research. In Week 2, most students regarded learning as an activity where students are taught by a teacher's highly structured direction and provision of knowledge. On the other hand, undergraduate research is a more open-ended, inductive and student-centred activity. They appeared to struggle to work out the research project due to this pedagogical gap. S2 said:

"There isn't a clear answer in advance, and no one will give an answer in doing research project. We have to build up hypothesis, and to verify it, we need to collect relevant data, and then analyse them in depth. So, research is totally different from learning. It puzzled me what to do in this research project." (S2, Group 1; Week 2, 2013)

The second factor is their values of interpersonal communication. Although students acknowledged that active participation was essential to their research project work, they tended to hesitate in active self-expression in the group. The major source of their reticence was not their fear of making mistakes itself, but an anxiety of whether they would disturb the collective work. For instance, since S6 highly valued intelligible explanation, he could rarely give uncertain information in the group. S6 said:

"I'm a poor talker by nature, and I don't want to bother others by sharing my uncertain idea. When I was not fully confident, I tended to hesitate to make contribution to the discussions." (S6, Group 2; Week 2, 2013)

The third factor is an understanding of research process. Practical difficulties during the research process include information searching, literature review, data collection and analysis. It was hard for them to obtain a clear image of what research is and what to do next during the course. S7 commented:

This was the first time to conduct qualitative research, so we couldn't even imagine what it is, and I didn't know what to do next in the research process. If we had to carry out research project by ourselves, I had no idea, like what I should do. (S7, Group 2, Final week, 2013)

The fourth factor is social relationships with tutors and peers. They sometimes felt that their participation was restricted by tutors' presence and instruction. S11 said:

"I think tutors had a strong presence. I know a tutor's opinion can be better than ours. I wasn't confident enough to express a thoughtful opinion in group discussions. So, sometimes I waited for tutor's suggestions rather than sharing my idea." (S11, Group 3, Week 2, 2014)

Furthermore, some students found it difficult to identify their own role in the group. For instance, S1 who was a more experienced member struggled to find a way to effectively contribute to the group work. S1 said:

"I have to find a suitable position in the group. It was difficult to identify my own role in this group. I felt other members were getting more independent, not relying on me. So, I need to think about how I can contribute to this group." (S1, Group 1, Week 2, 2013)

This interview excerpt shows that identity formation as a member is a key element of students' research activity, particularly in a collaborative learning context.

## Discussion

This study has drawn on phenomenography as research methodology to explore students' experiences in undergraduate research from the lens of study participants. Specifically, the focus of this study was on examining how research experiences informed students' perceptions of research and approaches to learning. Whilst all students were originally identified as aligning to a 'content-oriented' approach to studying, by the final week of the research project, ten out of 14 students in this study changed perceptions of research to a process-oriented view. By tracing changes in perception over time, data analysis revealed that, through participation in research project, their approaches to learning became qualitatively deeper, (-an inquiring mind, synthesis of knowledge, active participation, collaborative learning and reflective learning). Although four students' perceptions of research remained content-oriented, their strategic approaches to learning were also qualitatively changed. Although students took different approaches to learning in the undergraduate research, this study fully described the processes of changes in their perceptions of research and approaches to learning.

This study found that students' perceptions of research were (re-)formed through their actual research experiences, and these epistemological changes led to the adoption of deep approaches to learning in this course. The findings concurred with the previous studies which specified the key learning outcomes related to research skills.^28^^,^^29^ Specifically, two types of learning outcomes would be expected in research-based education.^28^

The first type is professional skills learning outcome which includes management of resources and time, self-directed learning, and communication skills. Students in this study could regard research as an activity with inquiring mind and mutual engagement within the context of "Research Experience" course. Thus, a given context, perception, and approaches to learning are reciprocally related.^23^

The second type is research skills learning outcome which includes critical appraisal and synthesis of evidence, formulating a research question and study design, data analysis and management.^28^^-^^30^ This learning outcome is fundamental not only to pursuing a research career but also to the routine practice and scholarly activity of all clinical professionals. Therefore, introduction of research-based education into the early undergraduate curriculum allows medical students to cultivate both research-specific skills and transferable skills, which are basically necessary for continuing professional development.^31^ The corroboration of this phenomenological study's findings with those from prior studies on undergraduate research in medical education indicate that collaborative research-based education should be implemented at the level of medical undergraduate curriculum as an essential springboard for becoming a medical professional. Findings from this study also demonstrate that students' awareness of the links between research and learning is an important outcome in undergraduate research.^32^ Through the research experience, students in this study could identify the vital link between research and learning. For instance, S2 mentioned in a final reflection that "*I feel research can include not only scientists' work but also our daily activity of learning*". As development of research skills is seen as an underlying principle in all education, learning through research is also pivotal to health professional development.^13^^,^^28^ Specifically, the inductive process of inquiry is closely connected with the principle of lifelong learning, clinical reasoning and EBM approach.^10^ Thus, research activity as an educational practice provides undergraduates with an opportunity not only to understand how the research process can contribute to the advancement of knowledge but also to enhance their research skills and active learning.

Research activity promotes students' active and reflective learning. In this study, some students were regularly reflecting on the progress of their research project and their own contributions to collective learning. Branch and Paranjape stated that "reflection leads to growth of the individual-morally, personally, psychologically, and emotionally, as well as cognitively".^33^ Learning journals and feedback are effective ways to further enhance their reflective learning in undergraduate research. In particular, provision of feedback from tutors is essential for promoting students' deep approach to learning.

Learners' cultural assumptions in relation to a collective activity are considered one of the elements that shape new learning process.^34^^,^^35^ In this study, students' values towards interpersonal communication were highly influential in their research experiences. Students addressed some difficulties in self-expression during the discussions, for instance, S6 said that *"I don't want to bother others by sharing my uncertain idea"*. Such a tendency came from not only their limited experience of student-initiated learning but also their values that prioritise a collective activity. However, as they recognised the importance of active self-expression in the group, the influence of their cultural assumptions gradually diminished. Medical educators need to understand that learning is shaped through students' ongoing participation, and they can adapt to the new learning context. Therefore, Japanese students, like other Asian students, cannot simply be categorised into a stable perception of quiet, passive and dependent learners.  Exploring the process of individuals' participation allows for better understanding variation in their ways of knowing, doing and being a member in a context of student-centred classroom.

An important aspect of facilitating students' active participation in research lies in keeping a balance between tutors' intervention and students' autonomy at each step of the research process.^5^^,^^36^ This study found that as the relationships between tutors and students in undergraduate research were socially dynamic, the roles of tutor needed to be defined according to students' prior research/learning experiences, the quality of research questions set by the students, and difficulties encountered during the research process.^4^^-^^7^ A better understanding of cultural, social or experiential factors that affect students' research activities are, thus, critical for enhancement of their active learning through undergraduate research.

## Conclusion

Medical education studies in undergraduate research to date have tended to focus on students' perceptions of research and curriculum descriptions of research-based education. This phenomenographic study revealed qualitative changes in students' perceptions of research and approaches to learning over time through observation of and reflection on their on-going participation in the research project. Although the sample size appeared to be relatively small, these findings could provide insights into the potential for undergraduate research in health professions education, which can further enhance students' deeper approach to learning and cultivate their basic skills necessary to continuing professional development. Furthermore, this evidence in this study can be a springboard for making more elaborative exploration of students' learning process in undergraduate research.

### Acknowledgments

We are grateful to the students who participated, and shared their experiences with us.

### Conflict of Interest

The authors declare that they have no conflict of interest.
